# Impact of digital Tai Chi interventions on the physical and mental health of older adults: a systematic review and meta-analysis

**DOI:** 10.3389/fpubh.2026.1831134

**Published:** 2026-06-04

**Authors:** Chaochao Zhao, Guifeng Hu, Dan Li, Yongshun Li

**Affiliations:** 1 Chongqing Business Vocational College, Chongqing, China; 2 Department of Basic Courses, Chongqing Medical and Pharmaceutical College, Chongqing, China; 3 Chongqing Bashu Science City Middle School, Chongqing, China; 4 Nanchong Vocational College of Culture and Tourism, Nanchong, Sichuan, China

**Keywords:** older adults, digital health intervention, human–computer interaction, mental health, meta-analysis, randomized controlled trials, Tai Chi

## Abstract

**Background and objective:**

Digital Tai Chi provides an expanding platform for geriatric rehabilitation. This study employs a meta-analysis to provide a preliminary evaluation of the integrated effects of digital Tai Chi interventions on physical function, psycho-cognitive health, and quality of life in older adults, with a specific focus on how varying levels of technological interaction modulate intervention efficacy.

**Methods:**

Electronic databases, including PubMed, Web of Science, Cochrane Library, and Embase, were searched through January 21, 2026. Randomized controlled trials (RCTs) investigating digital Tai Chi in older adults were included. Risk of bias was assessed using the RoB 2.0 tool, and effect sizes were synthesized using a random-effects model.

**Results:**

A total of 10 RCTs involving 907 participants were included. Meta-analysis results indicated that, in terms of psychological and cognitive outcomes, digital Tai Chi significantly improved global cognitive function (SMD = 0.64; 95% CI [0.09, 1.19]; *p* = 0.02) and alleviated depressive symptoms (SMD = −0.55; 95% CI [−1.03, −0.08]; *p* = 0.02). Notably, high-interaction technologies (e.g., AI feedback, VR) demonstrated superior effect stability in improving depression. Regarding physical function, improvements in the Timed Up and Go Test (TUGT) (MD = −0.63 s; 95% CI [−1.41, 0.16]; *p* = 0.12) and the Berg Balance Scale (BBS) (MD = 0.45; 95% CI [−0.94, 1.84]; *p* = 0.52) did not reach statistical significance. Subgroup analysis revealed that for quality of life and physical function indicators, the effects of digital Tai Chi were highly congruent with traditional face-to-face exercise, and significant benefits were primarily derived from comparisons with passive controls (e.g., routine care). GRADE assessment indicated that the quality of evidence ranged from “very low” to “moderate.”

**Conclusion:**

Digital Tai Chi demonstrate significant advantages in promoting cognitive function and mental health in older adults, with high-interaction technologies providing more stable psychological benefits. However, in the dimension of physical function remodeling, digital Tai Chi currently exhibits efficacy comparable to traditional exercise, without generating significant incremental advantages. This may be attributed to the limitations of existing technologies in providing deep proprioceptive feedback and high-precision motion correction. Future research should focus on the minimalist design of age-appropriate interfaces and develop high-precision interaction systems equipped with three-dimensional gravity and tactile feedback to overcome the “digital barriers” for older adults and enhance the precision of physiological interventions.

**Systematic review registration:**

This systematic review and meta-analysis has been registered in PROSPERO (www.crd.york.ac.uk/prospero), identifier CRD420261340491.

## 1 Introduction

Population aging has emerged as a major global public health challenge. The accompanying frailty, cognitive decline, and mental health issues significantly increase functional limitations and fall risks among the older adult(s) (1, 2). As a time-honored traditional Chinese mind–body exercise, Tai Chi has been widely proven to effectively improve postural control, muscle strength, and psychological resilience in older adults due to its emphasis on weight shifting, breath control, and meditative perception (3, 4). However, older adults often face complex biomechanical challenges when practicing Tai Chi. Furthermore, aging-related neurological degeneration not only impairs physical balance but is also frequently accompanied by complex physical and mental symptoms such as depression, anxiety, and executive dysfunction (5, 6). Therefore, seeking an integrated intervention strategy that simultaneously addresses physical functional remodeling and mental health promotion holds profound clinical significance for improving the quality of life of older adults and alleviating pressure on healthcare systems (7).

Although the evidence-based value of Tai Chi has been well recognized (8), traditional face-to-face teaching models face numerous barriers in practical implementation, including geographical distance constraints, a lack of professional coaching resources, transportation inconveniences, and poor long-term adherence due to participants’ limited mobility (9, 10). Particularly in the post-pandemic era, the demand for telehealth services has experienced explosive growth, driving the rapid development of Digital Health Interventions (DHIs) (11). The DHIs in this study encompass remote videoconferencing (e.g., Zoom), mobile applications (Apps), virtual reality (VR), augmented reality (AR), and intelligent feedback systems based on inertial measurement units (IMUs) (12). These technologies not only break physical spatial limitations but also provide a brand-new technical platform for personalized and precise Tai Chi rehabilitation through real-time motion correction, immersive environment simulation, and social interaction functions (13).

However, while digital Tai Chi interventions show great potential in the field of geriatric rehabilitation, significant knowledge gaps remain in existing research. First, previous systematic reviews have mostly focused on single-dimensional benefits, such as simple physical activity levels or basic balance indicators, lacking a systematic assessment of the “mind–body resonance” effect. This includes the interplay between physical functions like the Timed Up and Go Test and Berg Balance Scale, and deep psychological or cognitive health measured by the Montreal Cognitive Assessment and Geriatric Depression Scale under digital interventions (14, 15). Second, although high-interaction technologies such as AI-driven correction and VR immersion are becoming increasingly popular, high-quality quantitative evidence regarding their incremental effects compared to traditional video teaching or face-to-face models is still lacking (16). Furthermore, the specific efficacy and safety of digital Tai Chi interventions for older populations with different functional statuses, such as those with cognitive impairment, chronic pain, or healthy community-dwelling older adult(s), need further clarification (17).

Consequently, this study aims to comprehensively evaluate the integrated effects of various digital health technology-assisted Tai Chi interventions on the physical function, cognitive function, and psychological status of older populations through a systematic review and meta-analysis. This research focuses on exploring the differences between high-interaction digital technologies and traditional intervention models. The objective is to address existing gaps in evidence-based medicine and provide a scientific foundation for constructing precise, technology-driven management systems for geriatric mind–body rehabilitation.

## 2 Materials and methods

The protocol for this study has been registered with PROSPERO (Registration Number: CRD420261340491), which clearly defines the primary objectives, inclusion and exclusion criteria, intervention and control measures, as well as the planned primary and secondary outcome indicators. The implementation of this systematic review strictly followed the pre-registered protocol without significant deviation and adhered to the Preferred Reporting Items for Systematic Reviews and Meta-Analyses (PRISMA 2020) statement for implementation and reporting (18).

### 2.1 Search strategy

This study strictly followed the PRISMA guidelines, systematically searching the PubMed, Web of Science, Cochrane Library, Embase, Scopus, and CINAHL databases. The search period was set from the inception of each database up to January 21, 2026. The search strategy combined Medical Subject Headings with free-text terms, constructed across four dimensions: population (e.g., Aged, “older adults,” “older adult(s)”); exercise (e.g., “Tai Ji,” “Tai Chi,” “Mind–body exercise”); digital technology (e.g., “Virtual Reality,” “Mobile Applications,” “digital*,” “VR,” “AI,” “sensor-based”); and study type (e.g., “Randomized Controlled Trial”, “RCT”, “Clinical Trial”). Within each dimension, the logical operator “OR” was used for connection, while “AND” was employed to cross-search between dimensions. The retrieved literature records were imported into EndNote 21 software for de-duplication and unified management. Additionally, manual backward citation tracking of the included literature was performed to ensure the comprehensiveness of the search results. Detailed search terms are provided in Supplementary Text 1.

### 2.2 Inclusion and exclusion criteria

This study strictly established inclusion criteria based on the PICOS principles. The participants were limited to older adults aged 60 and above, encompassing healthy subjects, patients with chronic diseases, frail individuals, and those with mild cognitive impairment or dementia. Interventions were defined as digital technology-led Tai Chi practice, which included interactive modalities such as virtual reality, artificial intelligence feedback systems, remote videoconferencing, and sensor-assisted interventions. The control groups received traditional face-to-face Tai Chi, conventional exercises such as walking or stretching, health education, routine care, or the maintenance of baseline lifestyles. Outcome indicators were required to include at least one core data point among physical function, such as the Timed Up and Go Test and the Berg Balance Scale; psychological or cognitive status, including the Geriatric Depression Scale and the Montreal Cognitive Assessment; or health-related quality of life measured by the SF-36. Furthermore, only published randomized controlled trials were included. Exclusion criteria were defined as follows: non-randomized controlled studies; digital technology used solely for assessment rather than the intervention process; descriptive studies focusing only on system feasibility or usability; and studies where full texts were unavailable, data were missing, or those categorized as duplicate publications.

### 2.3 Data collection

Literature screening and data extraction were conducted independently by two researchers (the first and second authors). During the screening phase, a preliminary screening was first performed by reading titles and abstracts to exclude records that clearly did not meet the PICOS criteria. Subsequently, the full texts of potentially eligible studies were retrieved for in-depth re-screening, during which intervention details, participant characteristics, and data integrity were strictly verified, and reasons for exclusion were recorded in detail. A pre-designed standardized data extraction form was utilized, with two researchers independently extracting information from the 10 included randomized controlled trials and resolving any discrepancies through group discussion or consultation with a third-party expert. The extracted core content primarily included basic information such as the first author, publication year, and study location; participant characteristics including sample size per group, health status such as healthy, chronic disease, frailty, or cognitive impairment, and the specific age distribution of the experimental and control groups. Digital intervention details were also extracted, such as the technical form of Tai Chi including virtual reality, artificial intelligence feedback, and remote video, as well as practice frequency, session duration, total cycle length, and control group protocols. Outcome indicators comprised means and standard deviations at baseline and post-intervention for physical function measured by the Timed Up and Go Test and the Berg Balance Scale; psychological state assessed by the Geriatric Depression Scale; cognitive function evaluated via the Montreal Cognitive Assessment; and quality of life determined by the SF-36. The entire screening and extraction process was audited throughout by the third author. Any disagreements were resolved through collective discussion between the two researchers or by consulting the third author for a final decision. For studies with missing or inconsistently formatted data, such as those providing only medians and ranges, researchers attempted to ensure quantitative synthesis accuracy by obtaining original data through formula conversions or contacting the primary authors (19).

### 2.4 Risk of bias and certainty of evidence

Quality assessment of included studies was conducted using the Revised Cochrane risk-of-bias tool for randomized trials (RoB 2) (20). The evaluation covered five domains: bias arising from the randomization process, bias due to deviations from intended interventions, bias due to missing outcome data, bias in measurement of the outcome, and bias in selection of the reported result. Each domain was judged as “low risk,” “some concerns,” or “high risk,” culminating in an overall risk-of-bias determination for each study. The certainty of evidence was evaluated using the GRADE system, with downgrading assessments performed across five aspects: risk of bias, inconsistency, indirectness, imprecision, and publication bias (21). Evidence quality was categorized into four levels: high, moderate, low, or very low. Two reviewers independently completed the quality assessment, reaching consensus through discussion or expert consultation where necessary.

### 2.5 Data analysis

Statistical analysis was primarily performed using Review Manager 5.4 software. Since TUGT, BBS, MoCA, GDS, and SF-36 are all continuous variables, the pooled effect size was estimated by calculating standardized mean differences along with 95% confidence intervals. For TUGT and GDS indicators, a negative SMD value represents improvement, specifically a reduction in time or symptom severity. Statistical heterogeneity was assessed using the I^2^ test, where an I^2^ value of 25% or less indicates low heterogeneity, 25 to 50% indicates moderate heterogeneity, 50 to 75% indicates significant heterogeneity, and 75% or more indicates high heterogeneity (22). Although model selection is typically guided by the level of heterogeneity, this study employed a random-effects model for the meta-analysis of all outcomes as a conservative measure. This approach was intended to fully account for potential clinical heterogeneity arising from variations in study designs and participant characteristics, thereby ensuring a conservative estimation of the pooled results. Notably, to ensure the integrity of the data synthesis, clinical heterogeneity was managed through the aforementioned strict subgrouping framework rather than the exclusion of any large-scale trials. Furthermore, to rigorously manage clinical heterogeneity, subgroup analyses were employed to elucidate differences between studies. First, core subgroups were categorized based on the nature of the control group: the active control group referred to those receiving active exercise interventions, such as traditional face-to-face Tai Chi, walking, stretching, or balance training; the passive control group referred to those receiving routine care, health education, waitlist control, or maintaining their original lifestyle. Second preliminary exploratory subgroup analyses were conducted to identify potential trends associated with technological interaction features. Regarding the degree of digital interaction, studies utilizing automated and intelligent correction technologies—such as AI-based pose estimation, wearable sensor feedback, and virtual reality (VR) interaction—were categorized into the high-interaction group. Conversely, studies based on manual feedback delivered through media—such as online video guidance and real-time remote video interaction—were categorized into the low-interaction group. Given the limited number of included studies, these subgroup analyses were intended to identify tentative trends under different technological characteristics rather than to provide definitive statistical inferences (23). Publication bias was assessed using funnel plots and Egger’s test only when the number of studies included for a single outcome reached 10 or more; otherwise, these analyses were omitted. All statistical significance levels were defined as a *p*-value less than 0.05 (24).

## 3 Results

### 3.1 Study selection

**Figure 1:**
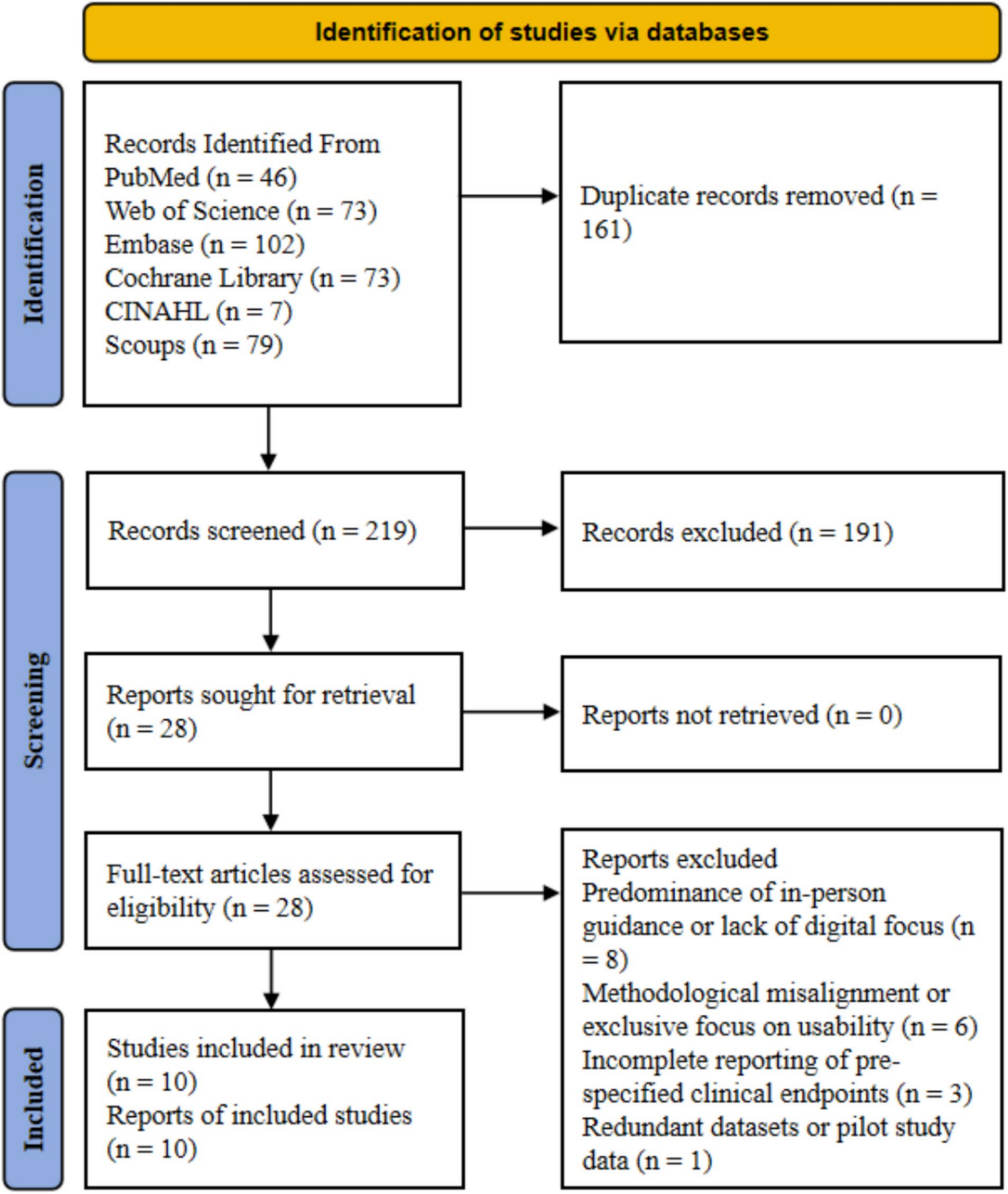
Preferred reporting items for systematic reviews and meta-analysis (PRISMA) study flow diagram.

Literature screening was conducted in strict accordance with the PRISMA 2020 guidelines, with the specific flow depicted in Figure 1. Through systematic searches across six core databases—PubMed, Web of Science, Cochrane Library, Embase, Scopus, and CINAHL—380 relevant records were initially identified. After removing duplicates (*n* = 165) using EndNote 21, the remaining 215 records underwent preliminary independent screening based on their titles and abstracts. During this phase, 179 articles were excluded for deviating from the research theme, such as non-older adult(s) populations or non-Tai Chi interventions. The remaining 36 reports proceeded to the full-text evaluation stage. Following a rigorous review by researchers, 18 documents were excluded for failing to meet the pre-specified criteria. The reasons for exclusion were distributed as follows: 8 articles were excluded because the intervention mode deviated from the digital core, primarily characterized by a high reliance on face-to-face offline guidance or digital technology serving only as an auxiliary demonstration; 6 articles were removed due to inconsistent methodological designs, including non-randomized controlled trials and usability studies limited to system feasibility assessments; 3 articles were excluded due to incomplete reporting of clinical endpoints, lacking core outcome indicators such as TUGT, MoCA, GDS, or SF-36; and 1 article was excluded for being a redundant dataset report or a preliminary exploratory study with an extremely small sample size. Ultimately, 10 randomized controlled trials (RCTs) were included for quantitative meta-analysis. The detailed screening process is illustrated in Figure 1.

### 3.2 Study characteristics

A total of 10 RCTs were ultimately included in this study (25–34), with publication years spanning from 2010 to 2025 (Table 1). The total included sample size was 907 cases, with 464 in the experimental group and 443 in the control group; individual study sample sizes ranged from 14 to 318 participants (27, 28). Subjects were primarily located in mainland China (*n* = 8) (27–35), the United States (*n* = 1) (25), and Taiwan, China (*n* = 1) (26), with a mean age range of 65.8 to 81.3 years (25, 32). Their health status encompassed healthy community-dwelling older adult(s) (27, 29, 30, 32, 34), patients with chronic diseases (28, 31), frail older adult(s) (25), and individuals with mild cognitive impairment (MCI) (33) or dementia (26). Regarding intervention details, the application of digital technology exhibited distinct interactive characteristics. High-interaction technologies (*n* = 7) utilized VR/AR systems, AI motion feedback (29), and IMU inertial sensors (30), emphasizing real-time calibration (26, 27, 33) and precise movement guidance (32, 34). Low-interaction technologies (*n* = 3) relied mainly on remote video conferencing (25, 28) or multimedia playback platforms (31). An intervention frequency of three times per week was most common, with intervention cycles ranging between 8 and 24 weeks (26, 32). Control group settings included active controls, such as traditional face-to-face Tai Chi (25) or walking training (34), and passive controls, such as routine care or health education (31). The primary outcome indicators covered multiple dimensions, including physical function (e.g., TUGT, BBS), psychological state (GDS), cognitive level (MoCA), and health-related quality of life (SF-36).

**Table 1:** Characteristics of the included studies.

Author (year)	Population/health status	Sample size (IG/CG)	Age (*M* ± SD, IG/CG)	Digital modality	Frequency	Duration	Period	Control group Type	Core outcomes
Wu et al. (2010) (25)	Frail older adult(s)	14/11	81.3 ± 4.5/81.1 ± 5.2	Tele-video	3 sessions/wk	60 min	12 wks	Traditional Tai Chi	TUGT, SF-36
Hsieh et al. (2018) (26)	Cognitive Impairment	31/29	76.4 ± 7.6/80.0 ± 7.5	VR (Kinect-based)	2 sessions/wk	60 min	24 wks	Passive control	MoCA
Chen et al. (2020) (27)	Healthy older adult(s)	7/7	68.2 ± 3.1/67.9 ± 2.8	Immersive VR	3 sessions/wk	30 min	8 wks	Traditional Tai Chi	TUGT, BBS
Li et al. (2023) (28)	Chronic diseases	159/159	70.1 ± 6.4/70.3 ± 6.1	Remote video	5 sessions/wk	50 min	12 wks	Routine care	TUGT, MoCA
Li et al. (2024) (30)	Healthy older adult(s)	30/30	66.8 ± 3.1/67.2 ± 3.5	IMU + TCNN system	3 sessions/wk	50 min	8 wks	Traditional Tai Chi	GDS
He et al. (2024) (29)	Community-dwelling	47/23	66.8 ± 5.2/67.1 ± 4.9	AI Feedback App	3 sessions/wk	45 min	12 wks	Traditional Tai Chi	TUGT, MoCA, SF-36
Qiu et al. (2024) (31)	Chronic diseases	40/40	67.0 ± 5.0 (Total)	Multimedia platform	5 sessions/wk	30 min	12 wks	Routine rehab	GDS, SF-36
Zhang et al. (2024) (32)	Community-dwelling	14/16	65.8 ± 4.1/66.2 ± 3.8	AI precise feedback	3 sessions/wk	40 min	8 wks	Traditional Tai Chi	GDS, SF-36
Zhu et al. (2025) (34)	Older adult(s) women	82/88	71.4 ± 5.3/70.9 ± 5.1	Interactive digital	3 sessions/wk	60 min	16 wks	Walking exercise	SF-36
Lo et al. (2025) (33)	MCI older adult(s)	40/40	72.5 ± 4.8/72.1 ± 4.5	AR/VR-assisted	2 sessions/wk	60 min	12 wks	Social control	TUGT, BBS GDS

IG, intervention group; CG, control group; M, mean; SD, standard deviation; TUGT, timed up and go test; MoCA, Montreal Cognitive Assessment; GDS, Geriatric Depression Scale; BBS, Berg Balance Scale; SF-36, 36-item Short Form Health Survey.

### 3.3 Risk of bias

**Figure 2:**
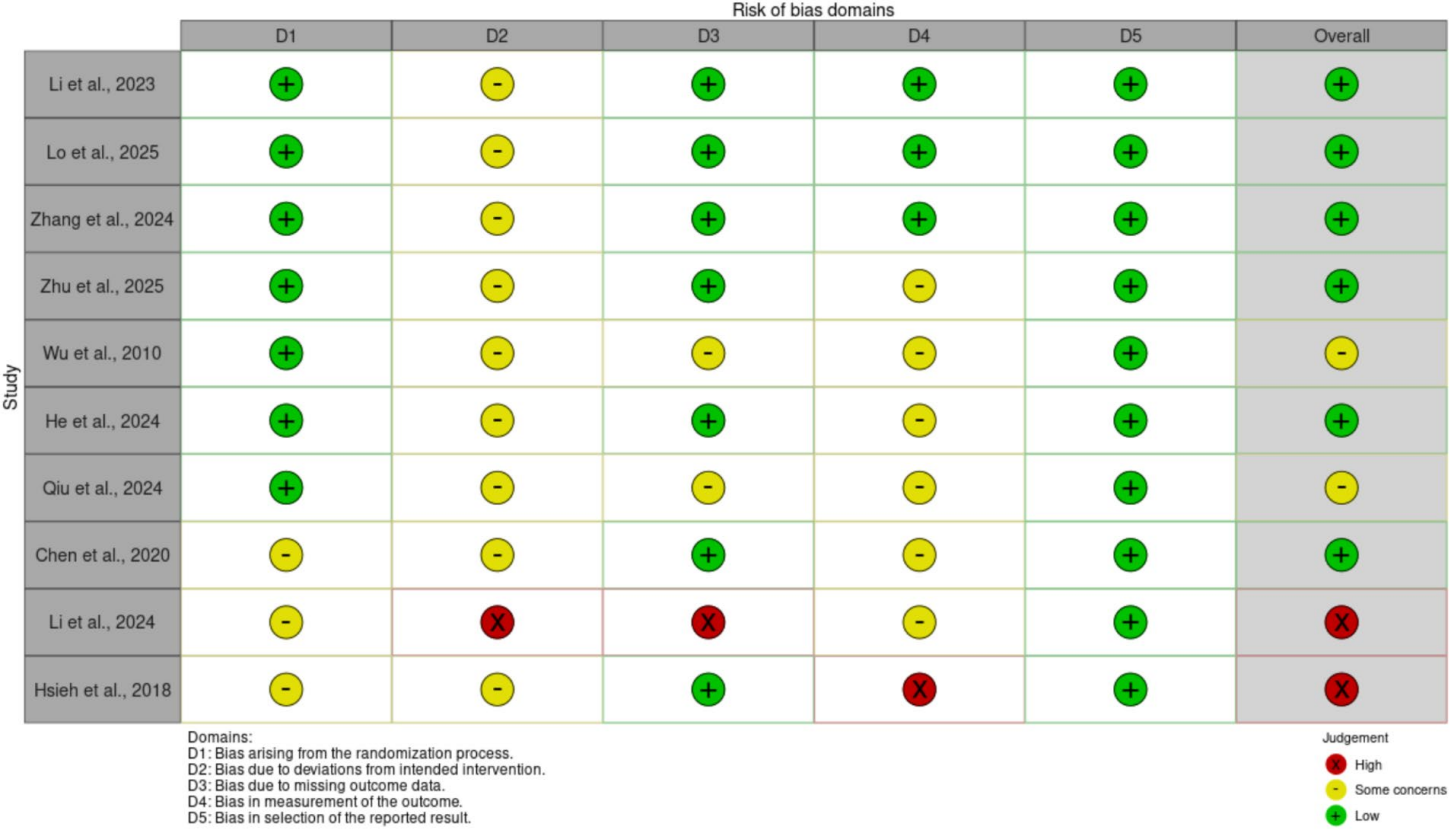
Risk of bias summary: review of the authors judgments about each risk of bias item for each included study.

**Figure 3:**
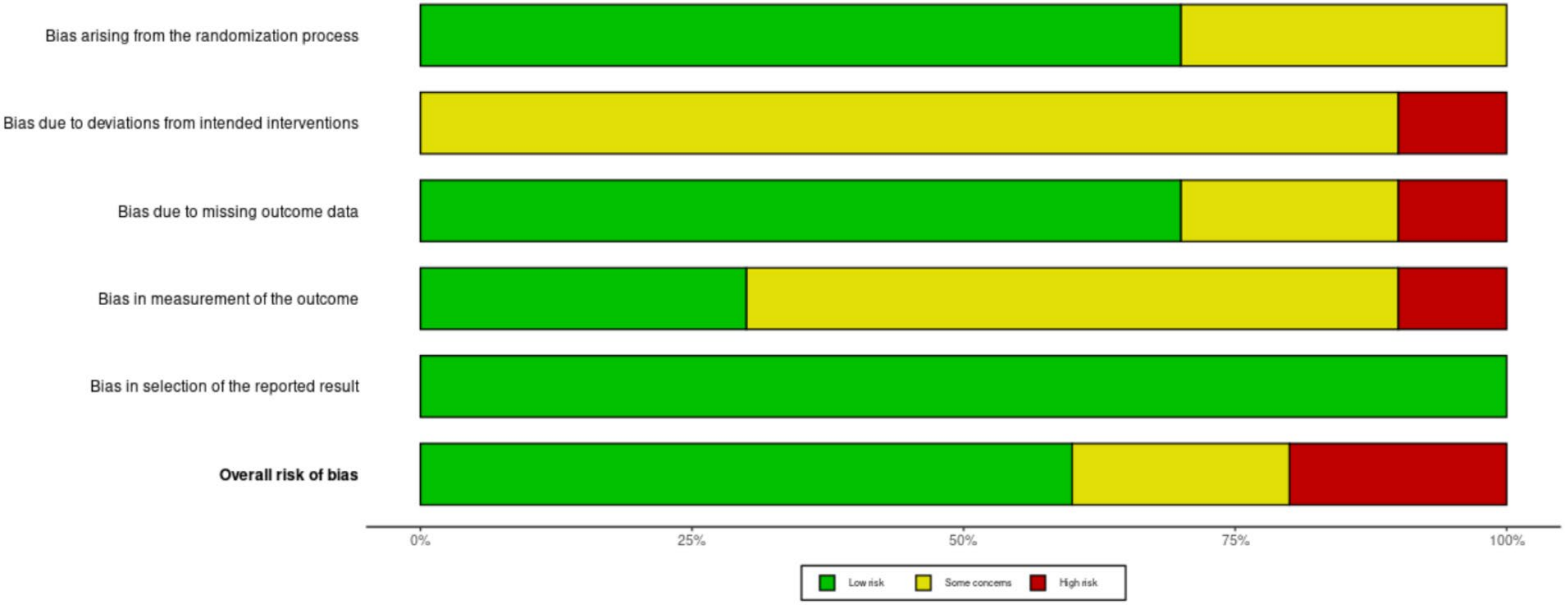
Risk of bias graph: review authors’ judgments about each risk of bias item, presented as percentage of included studies.

A multi-dimensional and systematic risk-of-bias assessment was conducted for the 10 included RCTs using the Cochrane RoB 2.0 tool, revealing some heterogeneity in methodological quality across different domains (Figures 2, 3). Overall, 60% (6/10) of the studies were rated as having a low risk of bias, including Li et al. (30), Lo et al. (33), Zhang et al. (32), and Zhu et al. (34), which demonstrated excellence in randomization protocols and the objectivity of outcome measurements. Another 20% of the studies raised “some concerns” (25, 31), while 20% were judged as having a high risk of bias (26, 30). In the domain of the randomization process (D1), 70% of the studies reported appropriate random sequence generation and allocation concealment mechanisms, with only rated as “some concerns” due to vague descriptions (26, 27, 30). In the domain of deviations from intended interventions (D2), participant blinding was difficult to achieve due to the inherent nature of exercise interventions; however, most high-quality studies mitigated implementation bias through rigorous intention-to-treat (ITT) analysis, with only Li et al. (30) judged as high risk due to a non-random loss to follow-up of up to 30% in the experimental group without imputation. Regarding the domain of missing outcome data (D3), data integrity remained high across most studies, except for Wu et al. (25), Li et al. (30) and Qiu et al. (31), which raised concerns due to high dropout rates in specific groups. The domain of outcome measurement (D4) was the primary source of bias; Hsieh et al. (26) was judged as high risk for explicitly admitting to not blinding assessors, while other studies involving subjective psychological scales (31) were marked as “some concerns” due to potential self-reporting bias from unblinded participants. Finally, in the domain of selection of the reported result (D5), all studies passed clinical trial registration checks, and no selective reporting behavior was found. In summary, while some studies were limited by objective constraints in blinding, the core evidence chain is supported by low-risk studies, ensuring the overall risk of bias remains within a controllable range and safeguarding the robustness of the meta-analysis conclusions.

### 3.4 Results

#### 3.4.1 Physical function indicators

**Figure 4:**
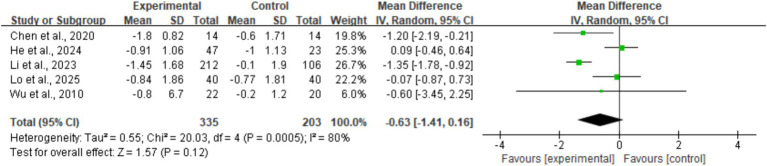
Forest plot of the effect of digital Tai Chi on TUGT in older adults.

**Figure 5:**
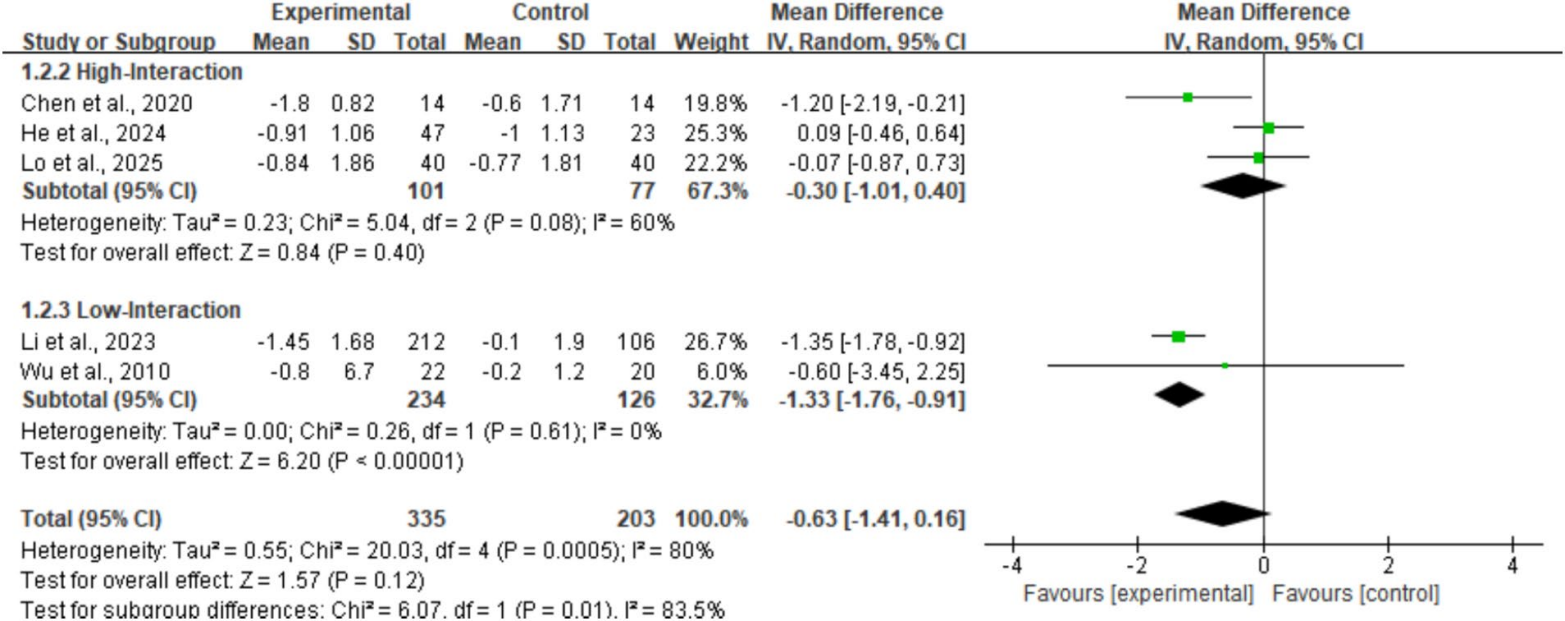
Forest plot of subgroup analysis for TUGT based on the level of technological interaction.

**Figure 6:**
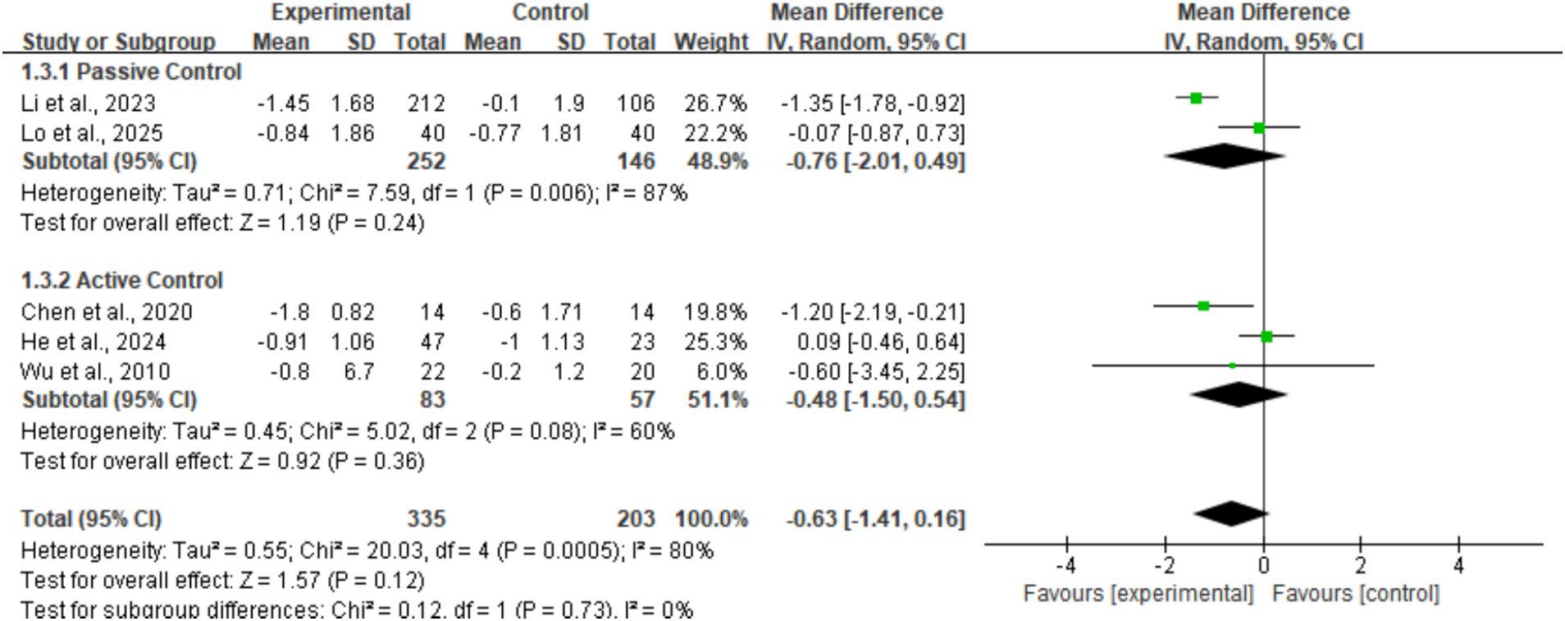
Forest plot of subgroup analysis for TUGT based on control group differences.

A total of 5 studies evaluated the effect of digital Tai Chi on TUGT (Figure 4). Initial meta-analysis indicated a decreasing trend in TUGT time within the intervention group, though the pooled difference did not reach statistical significance (MD = −0.63 s; 95% CI [−1.41, 0.16]; *p* = 0.12). A high level of heterogeneity was observed (I^2^ = 80%), which was systematically managed and explained through subgroup analysis to identify potential clinical moderators. When stratified by the level of technological interaction (Figure 5), the source of variance was clearly elucidated. The low-interaction group (2 studies) demonstrated a statistically significant improvement in TUGT (MD = −1.33 s; 95% CI [−1.76, −0.91]; *p* < 0.001) with absolute homogeneity (*I*^2^ = 0%). In contrast, the high-interaction group (3 studies) did not show a significant effect (MD = −0.30 s; 95% CI [−1.01, 0.40]; *p* = 0.40), exhibiting moderate within-subgroup heterogeneity (*I*^2^ = 60%). The test for between-subgroup differences confirmed a significant moderating effect of technological interaction levels (Chi^2^ = 6.07, df = 1, *p* = 0.01; *I*^2^ = 83.5%). The results indicated that the difference in effect sizes between the high-interaction and low-interaction groups was statistically significant. Subgroup analysis stratified by the nature of the control group showed (Figure 6) that the passive control group included 2 studies, and the pooled effect size was not statistically significant (MD = −0.76 s; 95% CI [−2.01, 0.49]; *p* = 0.24), with high within-subgroup heterogeneity (*I*^2^ = 87%). The active control group included 3 studies, and the pooled effect size was not statistically significant (MD = −0.48 s; 95% CI [−1.50, 0.54]; *p* = 0.36), with significant within-subgroup heterogeneity (*I*^2^ = 60%). Between-subgroup difference testing showed Chi^2^ = 0.12, df = 1, *p* = 0.73, and between-subgroup heterogeneity was *I*^2^ = 0%. The results suggested that the nature of the control group setting (active exercise vs. passive care) showed no statistically significant moderating effect on the TUGT index.

**Figure 7:**
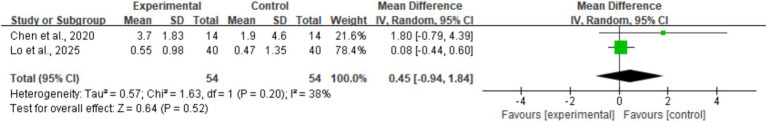
Forest plot of the effect of digital Tai Chi on BBS in older adults.

Two studies evaluated the effect of digital Tai Chi on the Berg Balance Scale (BBS) scores of older adults (Figure 7). The meta-analysis results showed that the difference in BBS score improvement between the intervention and control groups was not statistically significant (MD = 0.45; 95% CI [−0.94, 1.84]; *p* = 0.52). Moderate heterogeneity existed between the studies (*I*^2^ = 38%, *p* = 0.20).

#### 3.4.2 Cognitive and mental health indicators

**Figure 8:**
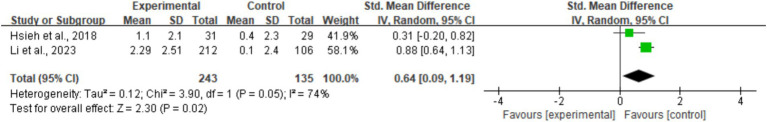
Forest plot of the effect of digital Tai Chi on MoCA in older adults.

Regarding cognitive function, two studies utilized the Montreal Cognitive Assessment (MoCA) for evaluation (Figure 8). The meta-analysis results demonstrated that the digital Tai Chi intervention group was significantly superior to the control group in enhancing global cognitive function in older adults (SMD = 0.64; 95% CI [0.09, 1.19]; *Z* = 2.30; *p* = 0.02). Significant high heterogeneity was present between the studies (*I*^2^ = 74%, *p* = 0.05).

**Figure 9:**
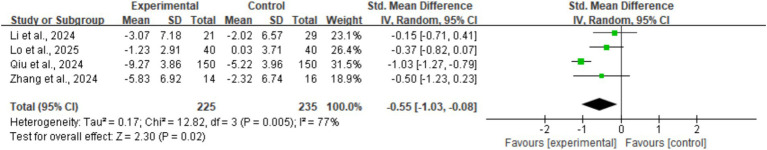
Forest plot of the effect of digital Tai Chi on GDS in older adults.

**Figure 10:**
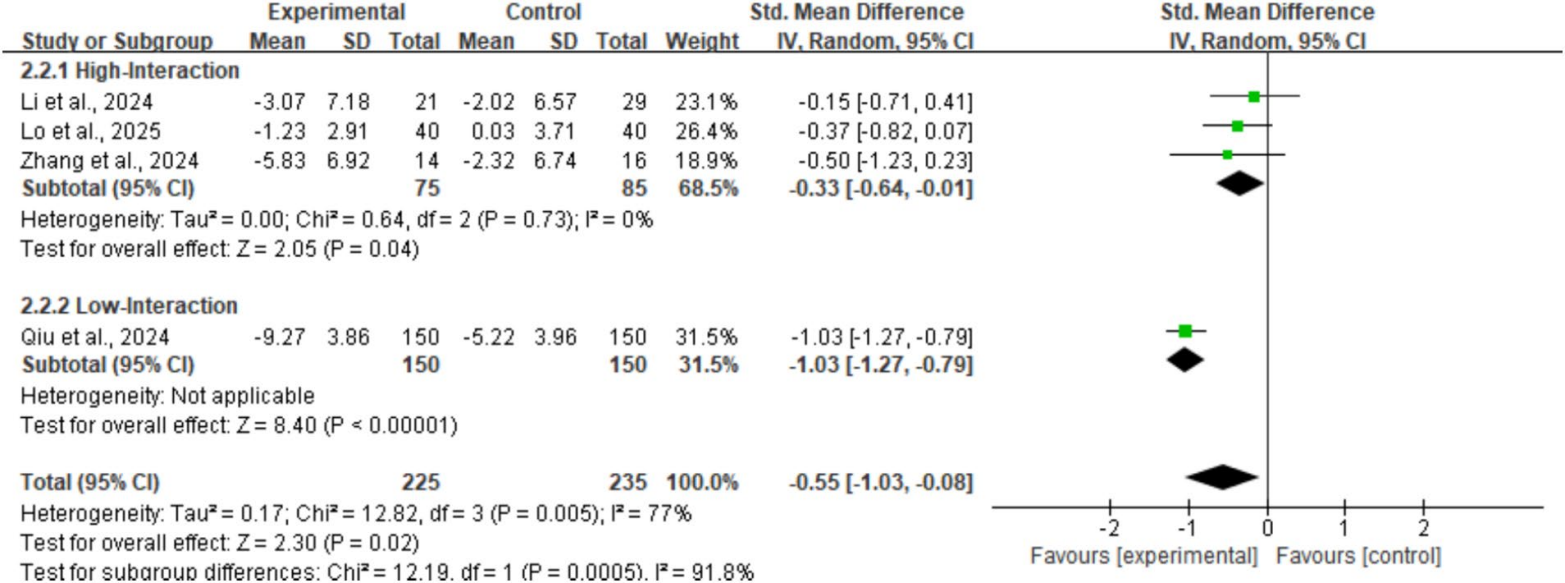
Forest plot of subgroup analysis for GDS based on the level of technological interaction.

**Figure 11:**
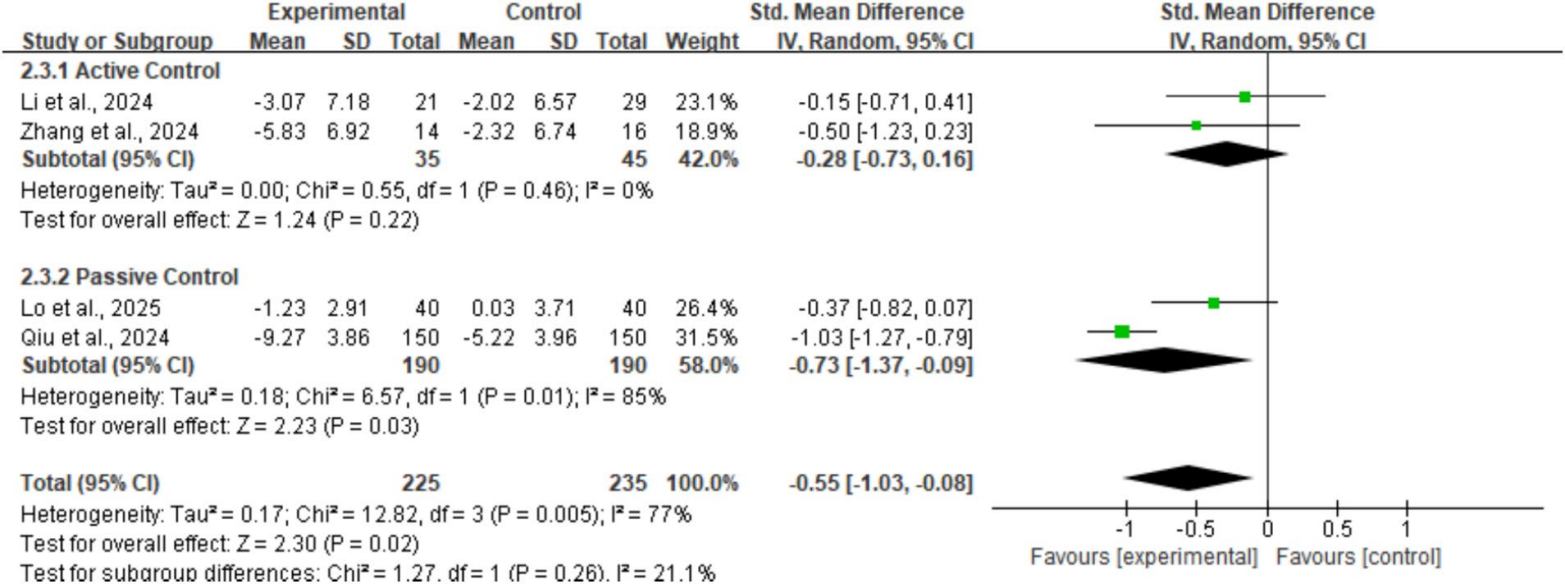
Forest plot of subgroup analysis for GDS based on control group differences.

A total of 4 studies evaluated the effect of digital Tai Chi on GDS in older adults (Figure 9). Meta-analysis showed that digital Tai Chi significantly alleviated depressive symptoms in older adults (SMD = −0.55; 95% CI [−1.03, −0.08]; *p* = 0.02). To address the significant heterogeneity observed in the full-sample synthesis (*I*^2^ = 77%), a strict subgroup analysis was performed to elucidate the moderating role of technological interaction levels (Figure 10). Subgroup analysis stratified by the level of technological interaction revealed, the pooled effect was statistically significant in the high-interaction group (SMD = −0.33; 95% CI [−0.64, −0.01]; *p* = 0.04; *I*^2^ = 0%), and also statistically significant in the low-interaction group (SMD = −1.03; 95% CI [−1.27, −0.79]; *p* < 0.001); between-subgroup difference testing showed Chi^2^ = 12.19, df = 1, *p* = 0.0005, *I*^2^ = 91.8%, indicating that the difference in effect sizes between different levels of technological interaction was statistically significant. Subgroup analysis stratified by the nature of the control group showed (Figure 11), the pooled effect was not statistically significant in the active control group (SMD = −0.28; 95% CI [−0.73, 0.16]; *p* = 0.22; *I*^2^ = 0%), whereas the pooled effect was statistically significant in the passive control group (SMD = −0.73; 95% CI [−1.37, −0.09]; *p* = 0.03; *I*^2^ = 85%); between-subgroup difference testing showed Chi^2^ = 1.27, df = 1, *p* = 0.26, *I*^2^ = 21.1%, indicating that the difference in effect sizes between the active and passive control groups was not statistically significant.

#### 3.4.3 Comprehensive quality of life indicators

**Figure 12:**
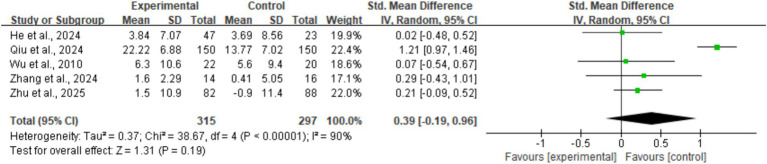
Forest plot of the effect of digital Tai Chi on SF-36 in older adults.

**Figure 13:**
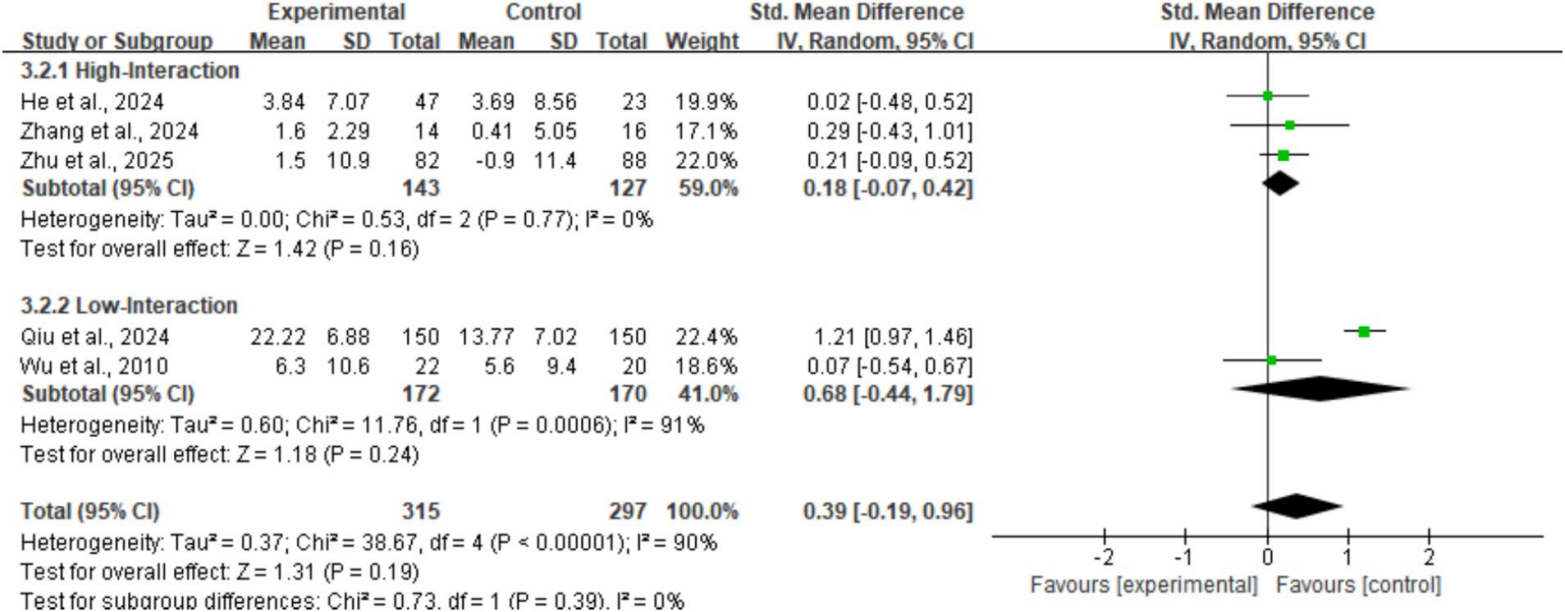
Forest plot of subgroup analysis for SF-36 based on the level of technological interaction.

**Figure 14:**
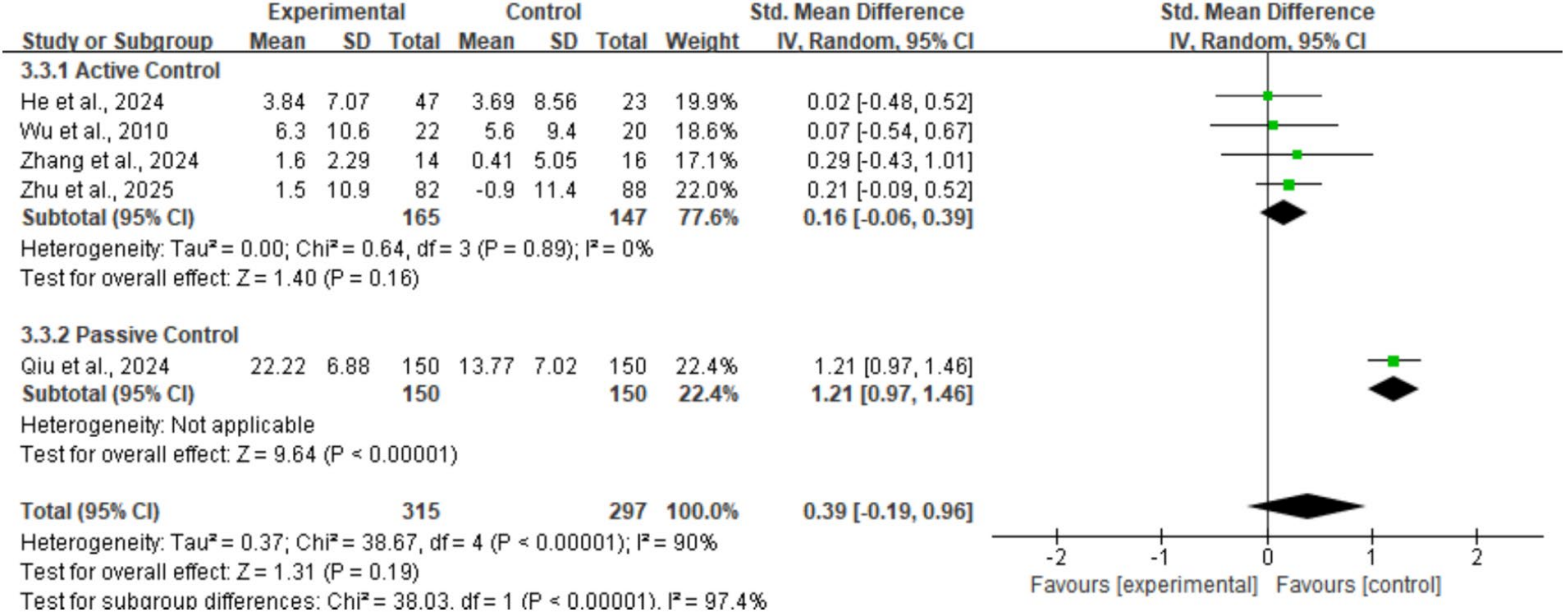
Forest plot of subgroup analysis for SF-36 based on control group differences.

A total of 5 studies evaluated the effect of digital Tai Chi on quality of life in older adults (Figure 12). Meta-analysis showed that the improvement in quality of life by digital Tai Chi was not statistically significant (SMD = 0.39; 95% CI [−0.19, 0.96]; *p* = 0.19), with extremely high heterogeneity (*I*^2^ = 90%). To address this, a strict subgrouping framework was employed to systematically manage and identify the clinical sources of variance. Subgroup analysis stratified by the level of technological interaction revealed (Figure 13), the pooled effect was not statistically significant in the high-interaction group (SMD = 0.18; 95% CI [−0.07, 0.42]; *p* = 0.16; *I*^2^ = 0%), and also not statistically significant in the low-interaction group (SMD = 0.68; 95% CI [−0.44, 1.79]; *p* = 0.24; *I*^2^ = 91%); between-subgroup difference testing showed Chi^2^ = 0.73, df = 1, *p* = 0.39, *I*^2^ = 0%, indicating that the difference in effect sizes between different levels of technological interaction was not statistically significant. Subgroup analysis stratified by the nature of the control group showed (Figure 14), the pooled effect was not statistically significant in the active control group (SMD = 0.16; 95% CI [−0.06, 0.39]; *p* = 0.16; *I*^2^ = 0%), whereas the pooled effect was statistically significant in the passive control group (SMD = 1.21; 95% CI [0.97, 1.46]; *p* < 0.001); between-subgroup difference testing showed Chi^2^ = 38.03, df = 1, *p* < 0.00001, *I*^2^ = 97.4%, indicating that the difference in effect sizes between the active and passive control groups was highly statistically significant.

### 3.5 Publication bias

For all core outcome indicators involved in this study, including TUGT, BBS, MoCA, GDS, and SF-36, the number of original articles included was less than 10. This did not meet the minimum methodological requirements for conducting quantitative assessments of publication bias, such as funnel plot analysis or Egger’s test. Therefore, statistical testing for publication bias was not performed for each outcome indicator in this study. The pooled analysis results of the relevant indicators should be interpreted cautiously in conjunction with their sample sizes and certainty of evidence.

### 3.6 Quality of evidence

In this study, the GRADE (Grading of Recommendations Assessment, Development, and Evaluation) system was employed to evaluate the certainty of evidence for five core outcome measures (Table 2). The results indicated that the quality of evidence for digital Tai Chi interventions in improving depressive symptoms (GDS), the Timed Up and Go Test (TUGT), and the Berg Balance Scale (BBS) was categorized as “Moderate.” This downgrading was primarily attributed to significant clinical and statistical heterogeneity across studies, as well as imprecision resulting from the limited number of included trials. The certainty of evidence for Health-Related Quality of Life (SF-36) was rated as “Low,” largely due to extreme heterogeneity (I^2^ = 90%) stemming from diverse control group designs and a lack of statistical significance in the pooled effect size. The evidence for the Montreal Cognitive Assessment (MoCA) was rated as “Very Low,” closely associated with a high risk of bias in several included studies, substantial heterogeneity, and small sample sizes. Furthermore, given that the number of studies included for each outcome measure was fewer than 10, a quantitative assessment of publication bias (e.g., funnel plots) was not conducted. To ensure transparency, all eligible studies were included in the synthesis, and the associated effect sizes should be interpreted with appropriate caution in light of the observed clinical heterogeneity.

**Table 2:** GRADE evidence profile for the impact of digital Tai Chi intervention on the physical and mental health of older adults.

Outcomes	No. of studies (design)	Risk of bias	Inconsistency	Indirectness	Imprecision	Publication bias	Certainty of evidence
TUGT	5 (RCT)	Not serious	Serious (−1)a	Not serious	Not serious	Not evaluated e	Moderate
BBS	2 (RCT)	Not serious	Not serious	Not serious	Serious (−1)b	Not evaluated e	Moderate
MoCA	2 (RCT)	Serious (−1)c	Serious (−1)a	Not serious	Serious (−1)b	Not evaluated e	Very low
GDS	4 (RCT)	Not serious	Serious (−1)a	Not serious	Not serious	Not evaluated e	Moderate
SF-36	5 (RCT)	Not serious	Very serious (−2)d	Not serious	Not serious	Not evaluated e	Low

a. Inconsistency: High *I^2^* value, with significant clinical heterogeneity observed in participant baselines and control group settings among the included studies; b. Imprecision: sample size was small and the confidence interval crossed the line of no effect or was wide; c. Risk of bias: included studies contained literature with a high risk of bias; d. *I^2^* was as high as 90%, reflecting significant clinical heterogeneity primarily arising from the diversity in control group designs; e. Publication bias: a quantitative assessment via funnel plots was not performed due to the limited number of studies (*k* < 10). However, all eligible studies were included in the synthesis to ensure the transparency of the results. The potential risk of publication bias cannot be entirely ruled out.

## 4 Discussion

### 4.1 Summary of research findings

The present study included 10 randomized controlled trials involving a total of 907 older adult(s) participants. Meta-analysis results showed that digital Tai Chi intervention was statistically significant in improving global cognitive function and alleviating depressive symptoms among older adults; whereas for the timed up and go test, Berg Balance Scale, and quality of life outcomes, no statistically significant pooled differences were observed between the intervention and control groups. Subgroup analyses were utilized to systematically manage and provide explanatory attributions for the substantial clinical heterogeneity observed across studies. Regarding the level of technological interaction, the high-interaction subgroup for mental health (GDS) exhibited extremely high effect stability, with a superior improvement effect compared with the low-interaction subgroup; for physical function (TUGT), the effect size was significantly moderated by technological interaction level, with the low-interaction subgroup showing significant improvement and no within-subgroup heterogeneity; for quality of life (SF-36), technological interaction level showed no statistically significant moderating effect.

Regarding the nature of the control group, the pooled effects of digital Tai Chi versus active control groups were not statistically significant for both quality of life and depressive symptoms, and within-subgroup heterogeneity was notably reduced, indicating that digital Tai Chi yielded comparable effects to traditional in-person exercise. The reduction in within-subgroup heterogeneity further validates that the overall statistical variance was primarily driven by clinical design differences rather than study quality, and its statistically significant beneficial effects were mainly derived from comparisons with passive control groups; for TUGT, the nature of the control group showed no significant moderating effect. According to the GRADE system assessment, the quality of evidence for all current outcomes ranged from “very low” to “moderate.” Downgrading was mainly attributed to significant clinical heterogeneity stemming from diverse control settings, and imprecision due to limited sample sizes for some outcomes. Quantitative assessment of publication bias was not performed in this study because fewer than 10 studies were included for each outcome measure. In summary, digital Tai Chi shows certain advantages in improving cognitive and mental health, and demonstrates effects similar to traditional exercise interventions on physical function and quality of life. However, the quantitative findings still require further verification by more large-sample, high-quality RCTs.

### 4.2 Comparison with previous research and analysis of causes

The meta-analysis results of this study indicate that digital Tai Chi interventions demonstrate statistical significance in improving cognitive function and alleviating depressive symptoms in older adults, which aligns with recent findings suggesting that online exercise interventions improve physical and mental health (35). This phenomenon may imply that digital mind–body exercises possess certain application value in mental health maintenance. However, the present study shows that the improvements in TUGT and BBS through digital Tai Chi did not reach statistical significance, contrasting with the conclusions widely reported in traditional face-to-face Tai Chi research (10). This inconsistency may reflect that when digital interventions are applied to frail older adult(s) or patients with cognitive impairment who have weaker baseline levels, there may be inherent barriers to the subjects’ understanding and execution of digital commands (36). Regarding the significant heterogeneity observed in physical function indicators, specifically an *I*^2^ of 80% for TUGT, the clinical heterogeneity may primarily stem from the vast span of the subjects’ functional status. Participants ranged from healthy community-dwelling older adult(s) to those with mild cognitive impairment or dementia, potentially leading to tiered differences in their ability to recruit complex movements. Furthermore, clinical heterogeneity is manifest in the heterogeneous nature of the control group settings. While some studies utilized routine care as a passive control, the majority employed traditional face-to-face Tai Chi as an active control. Such discrepancies in control group efficacy inevitably diluted the relative advantage of digital interventions during the pooling of effect sizes, leading to more conservative quantitative synthesis results. This suggests that the clinical value of digital Tai Chi may lie more in serving populations who lack access to professional offline guidance, rather than demonstrating a definitive superiority over traditional face-to-face modes in direct comparisons. Additionally, the significant spectrum of technological interaction—ranging from simple multimedia guidance to complex sensor-based feedback systems—may contribute to conflicting effect sizes across studies. Subgroup analyses further clarified that the high heterogeneity in TUGT findings was primarily driven by clinical variations in control settings; however, even with all eligible studies synthesized, the current evidence appears insufficient to conclude that digital Tai Chi is superior to control groups in enhancing physical mobility. Furthermore, the control groups in both trials were set as non-exercise-based routine care. Subgroup analysis showed that in terms of quality of life and physical function outcomes, the effect sizes of digital Tai Chi and the active control group (traditional in-person exercise) were highly consistent, and within-subgroup heterogeneity was significantly reduced to 0%. This suggests that the clinical value of digital Tai Chi may be more reflected as an effective supplement to the traditional in-person model, with similar efficacy to offline guidance in maintaining physiological function dimensions, rather than significantly outperforming the traditional model in direct comparisons. The considerable variation in the level of digital technological interaction was also a key factor contributing to conflicting effect sizes. Subgroup analysis confirmed that the level of technological interaction exerted a significant moderating effect on both physical function and mental health. For the TUGT outcome, low-interaction technology exhibited a more distinct improvement effect; whereas for the GDS outcome, the high-interaction group demonstrated extremely high effect stability. This divergence suggests that different levels of digital interaction may drive physiological adaptation and psychological engagement through distinct pathways: while low-interaction modes may facilitate physical execution through simpler guidance, high-interaction systems appear more effective in sustaining psychological immersion and emotional regulation.

From the perspective of biomechanics and neuroscience, it is speculated that the potential drive of digital Tai Chi on cognitive function may stem from its high cognitive load exercise characteristics. Tai Chi involves complex sequence learning and breathing control; when combined with real-time prompts from platforms such as AI feedback, it could theoretically stimulate the executive functions of the prefrontal cortex (37). On a psychological level, VR and AI technologies may enhance the enjoyment and social presence of practice through gamified interfaces, thereby increasing adherence to some extent and driving improvements in depressive symptoms (38). In particular, high-interaction systems equipped with real-time feedback mechanisms may more effectively mobilize participants’ emotion regulation abilities through the reinforcement of a “perception–action–closed-loop” process, compared with traditional one-way video guidance. In contrast, the improvement of balance ability may highly depend on precise plantar pressure feedback and fine-tuning of center-of-gravity trajectories (27, 29). If most current digital interventions continue to focus on visual guidance while lacking the physical correction and three-dimensional proprioceptive feedback provided by professional coaches in traditional face-to-face instruction, it may be difficult to produce the expected incremental effects in the physical function remodeling dimension (39, 40). Regarding the phenomenon where significant advantages in physical function improvement have not yet emerged, we infer that the core reason may lie in a certain lack of depth in sensory-motor integration within digital interventions. On one hand, the irreplaceable nature of physical feedback may be of key significance; real-time manual correction and resistance guidance provided by professional coaches in traditional teaching are often crucial for correcting subtle deviations during center-of-gravity transfer. In contrast, current digital platforms mostly focus on visual guidance and lack such deep tactile and proprioceptive feedback, which may lead subjects to merely imitate movements visually without truly reshaping deep postural control abilities (41). On the other hand, the improvement of balance ability is highly dependent on precise plantar pressure distribution and fine-tuning of center-of-gravity trajectories. If subjects only practice through two-dimensional screens or VR environments with insufficient depth perception information, it may be difficult to establish accurate proprioceptive mapping in three-dimensional physical space (42). Furthermore, digital interventions may have limitations in precision or feedback delays when identifying complex biomechanical errors such as knee valgus or pelvic misalignment, which may also limit the effectiveness of their perception-action-correction closed loop, making it difficult to produce physiological adaptation effects that significantly surpass traditional exercise (43). This inference was indirectly supported by our subgroup analysis: when digital Tai Chi was compared with passive controls (non-exercise), its significant beneficial effects indicated its fundamental value as an exercise intervention; however, when compared with active controls with advantages in physical correction, existing technological approaches have not yet yielded obvious incremental benefits. In summary, although digital technology has broken the physical limits of geographical space, existing digital means may not have yet reached the technical threshold required to produce significant incremental effects in dimensions of physical function remodeling that involve high physical interaction and precise proprioceptive feedback (44).

### 4.3 Research limitations

This study provides a preliminary exploration of the effects of digital modes on Tai Chi’s health benefits, yet several limitations regarding clinical application and methodological rigor remain. First, since the number of studies included for each outcome measure was fewer than 10, quantitative tests for publication bias, such as funnel plots, were not conducted. Despite an extensive literature search, the risk of potential publication bias due to limited study scale remains; thus, the interpretation of pooled effect sizes should remain cautious. Additionally, constrained by the total sample size, the subgroup analyses based on technological interaction levels are exploratory in nature and possess limited statistical power. While the results exhibit a certain trend of effect gradients, they are insufficient to support definitive statistical inferences. Second, there is a conflict between the universality of technological interaction design and digital barriers. Although this study explored differences in effects across various interaction modes, these technical solutions are largely based on standardized design logic and fail to fully account for the digital barriers commonly encountered by older adults (45, 46). Moreover, the quantitative data synthesis faced significant clinical heterogeneity: participants ranged from healthy community-dwelling older adult(s) to those with cognitive or physical impairments, and control groups included both “active exercise” and “passive care” modes. While this clinical non-homogeneity was systematically managed through subgroup analyses to enhance transparency, the inherent diversity in population baselines and control protocols still necessitates a cautious interpretation of the overall pooled effect sizes. In practice, significant individual gaps exist among the older adult(s) regarding the cognitive costs of digital interfaces, hand-eye coordination, and the ability to comprehend complex feedback instructions. This study did not simulate how the operational frustration triggered by technical intervention might conversely weaken the original physical and mental regulatory benefits of Tai Chi, thereby limiting the generalizability of the conclusions to older populations with low digital literacy. For instance, the attrition rate as high as 30% in the experimental group of Li et al. (28) partially validates the negative impact of operational frustration on intervention adherence. Second, the implicit stigma triggered by digital monitoring mechanisms remains a concern. In quantifying physical and mental impacts, this research primarily focused on the positive dimensions of technical feedback while overlooking the possibility that real-time monitoring of movement precision might constitute a digital gaze (47). For some older adults, the digital presentation of physical functions often forces a direct confrontation with the reality of their own aging, a perception that can easily induce stigma or evaluation anxiety (48). This psychological pressure may act as a confounding variable affecting participants’ emotional states and autonomic nervous system responses during practice; however, current quantitative models find it difficult to fully isolate the impact of this psychological resistance on physiological indicators.

In addition, there were limitations in the simulation of real-life scenarios within the experimental settings. Although subgroup analysis explained the heterogeneity across studies to some extent, the included studies were mainly conducted under controlled experimental conditions, lacking ecological validity verification in natural living environments such as home settings. In real-world settings, the effectiveness of digital Tai Chi interventions depends not only on the technology itself but also on complex influences such as household physical space, equipment maintenance capabilities, and social support environments. This study did not fully investigate how technological interaction performs in the presence of multi-task interference or in the absence of professional assistance, leading to uncertainty when translating results into normalized community and home applications. Although this study achieved interdisciplinary coverage through WoS and Scopus, it did not specifically search computer science databases such as ACM or IEEE, potentially omitting technical studies focused on interaction prototype validation rather than clinical randomized controlled trials (RCTs). Finally, there is a lack of longitudinal investigation into the long-term efficacy and psychological fatigue associated with technical interventions. Current conclusions are mostly based on short-to-medium-term intervention experiments and fail to observe the long-term adaptation process of older adults to specific digital interaction modes. As intervention periods extend, older adult(s) practitioners may shift from an initial novelty-driven motivation to technical fatigue caused by cumbersome operations, and may even lose their ability to perceive proprioception due to over-reliance on digital feedback (49). The participant dropout caused by such technology fatigue suggests that future research should focus on the minimalist design of interaction logic. Future studies urgently need to construct multi-year longitudinal tracking systems to clarify the decay patterns and psychological dependency mechanisms involved in the psychosomatic remodeling of older adults via digital interventions.

### 4.4 Practical significance and enlightenment

Based on the preliminary findings of the subgroup analysis on technological interaction modes, this study suggests that the design of future age-appropriate fitness equipment requires a paradigm shift from “action-standard orientation” to “emotional-experience orientation.” In future product development, systems might incorporate more flexible error-correction logic, dynamically adjusting interaction intensity according to the practitioner’s real-time physiological feedback and operational proficiency. This adaptive guidance mechanism could help alleviate stress responses in older adults during their initial contact with digital technology, thereby mitigating the negative impact of digital barriers on intervention effectiveness at the source. At the psychological support level, our analysis hints at potential pathways for neutralizing the “stigma” associated with digital monitoring. The promotion of digital Tai Chi should perhaps increasingly adopt non-invasive (imperceptible) data collection technologies and attempt to transform dry quantitative indicators into visual feedback enriched with cultural connotations or gamified narratives. Such a design approach could help reduce the anxiety of “digital gaze” among older practitioners, transforming technological intervention from cold monitoring into positive psychological incentive, thereby enhancing the self-efficacy of older adults in digital health environments.

From the perspective of social service models, the popularization of digital Tai Chi may drive the evolution of community sports management toward an integrated model of “digital intelligence and humanistic care.” In future practical scenarios, the differentiated data collected by digital devices could provide personalized intervention benchmarks for community physicians or volunteers. By synergizing precise online feedback with offline social support, it may be possible to bridge the gap in emotional communication inherent in standalone technological interactions, providing a reference framework for building low-cost, high-efficiency chronic disease management systems for the older adult(s). Furthermore, the preliminary findings of this study may serve as a reference for the formulation of relevant industry standards. It is speculated that when evaluating digital sports products for the older adult(s), governments and industry associations might focus not only on functional indicators but also incorporate the degree of age-appropriate design and mental health protection mechanisms into the audit scope. Such a policy-level orientation could encourage more enterprises to involve geriatric psychology research during the R&D stage, thereby promoting the deep integration of digital technology with traditional exercise modalities on a macro scale and providing novel solutions for national health issues amidst an aging population.

## 5 Conclusion

Through a systematic review and meta-analysis of existing randomized controlled trials, the present study indicates that digital Tai Chi intervention may show a certain beneficial trend in improving global cognitive function and depressive symptoms among older adults; whereas for physical function (TUGT, BBS) and overall quality of life (SF-36) outcomes, the intervention group has not yet demonstrated a statistically definitive advantage over the control group. Subgroup analyses preliminarily revealed potential sources of heterogeneity across studies. For physical function and quality of life outcomes, digital interventions showed relatively high convergence in effects with active exercise control groups, suggesting their potential feasibility as an alternative to traditional exercise. For mental health outcomes, high-interaction technologies with real-time closed-loop mechanisms exhibited a relatively more stable effect trend. In summary, although preliminary evidence suggests the application value of digital Tai Chi in cognitive and psychological interventions, its superiority or incremental effect relative to traditional modalities remains inconclusive due to the small sample size of included studies and clinical heterogeneity. Limitations of digital models in aspects such as deep proprioceptive feedback and overcoming digital barriers among older adults may be key factors constraining their efficacy in improving physical function. Given the generally low certainty of evidence for current outcome measures, the pooled results should be regarded as preliminary exploration in this field. Large-sample and long-term clinical trials based on different technological interaction logics are urgently needed in the future to further clarify the actual effects of digital Tai Chi on physical and mental health in older adults.

## Data Availability

The original contributions presented in the study are included in the article/Supplementary material, further inquiries can be directed to the corresponding author.
