# Supplementary material for: Impact of digital Tai Chi interventions on the physical and mental health of older adults: a systematic review and meta-analysis
Source: Front Public Health. 2026 Jun 4;14:1831134. doi: 10.3389/fpubh.2026.1831134 (PMC13275682; doi:10.3389/fpubh.2026.1831134)
Supplement: Supplementary file 1 [file Data_Sheet_1.pdf]

Supplementary Text 1 Search Strategies

1. PubMed:

search strategy:

#1 (Aged[MeSH] OR aged[tiab] OR "older adults"[tiab] OR "older people"[tiab] OR elderly[tiab] OR senior*[tiab] OR aging[tiab])

#2 ("Tai Ji"[MeSH] OR "Tai Chi"[tiab] OR Taiji*[tiab] OR Taichi[tiab] OR "Tai Chi Chuan"[tiab] OR "Mind-body exercise"[tiab])

#3 ("Virtual Reality"[MeSH] OR "Mobile Applications"[MeSH] OR "Telemedicine"[MeSH] OR "Telerehabilitation"[MeSH] OR digital*[tiab] OR "virtual reality"[tiab] OR VR[tiab] OR "augmented reality"[tiab] OR AR[tiab] OR "mixed reality"[tiab] OR MR[tiab] OR APP[tiab] OR smartphone[tiab] OR "mobile health"[tiab] OR mHealth[tiab] OR "remote intervention"[tiab] OR "tele-rehabilitation"[tiab] OR exergame*[tiab] OR "video game*"[tiab] OR "sensor-based"[tiab] OR "artificial intelligence"[tiab] OR AI[tiab] OR wearable*[tiab])

#4 ("Randomized Controlled Trial"[MeSH] OR "randomized controlled trial"[tiab] OR random*[tiab] OR placebo[tiab] OR "clinical trial"[tiab] OR RCT[tiab])

#5 #1 AND #2 AND #3 AND #4

2. Web of Science:

search strategy:

#1 TS=("aged" OR "older adults" OR "older people" OR "elderly" OR "senior*" OR "aging")

#2 TS=("Tai Ji" OR "Tai Chi" OR "Taiji*" OR "Taichi" OR "Tai Chi Chuan" OR "Mind-body exercise")

#3 TS=("Virtual Reality" OR "Mobile Applications" OR "Telemedicine" OR "Telerehabilitation" OR "digital*" OR "virtual reality" OR "VR" OR "augmented reality" OR "AR" OR "mixed reality" OR "MR" OR "APP" OR "smartphone" OR "mobile health" OR "mHealth" OR "remote intervention" OR "tele-rehabilitation" OR "exergame*" OR "video game*" OR "sensor-based" OR "artificial intelligence" OR "AI" OR "wearable*")

#4 TS=("randomized controlled trial" OR "random*" OR "placebo" OR "clinical trial" OR "RCT")

#5 #1 AND #2 AND #3 AND #4)

3. Embase:

search strategy:

#1 'aged'/exp OR 'aged':ab,ti OR 'older adults':ab,ti OR 'older people':ab,ti OR 'elderly':ab,ti OR 'senior*':ab,ti OR 'aging':ab,ti

#2 'tai ji'/exp OR 'tai chi':ab,ti OR 'taiji*':ab,ti OR 'taichi':ab,ti OR 'tai chi chuan':ab,ti OR 'mind-body exercise':ab,ti

#3 'virtual reality'/exp OR 'mobile application'/exp OR 'telemedicine'/exp OR 'telerehabilitation'/exp OR 'digital*':ab,ti OR 'virtual reality':ab,ti OR 'vr':ab,ti OR 'augmented reality':ab,ti OR 'ar':ab,ti OR 'mixed reality':ab,ti OR 'mr':ab,ti OR 'app':ab,ti OR 'smartphone':ab,ti OR 'mobile health':ab,ti OR 'mhealth':ab,ti OR 'remote intervention':ab,ti OR 'tele-rehabilitation':ab,ti OR 'exergame*':ab,ti OR 'video

game*':ab,ti OR 'sensor-based':ab,ti OR 'artificial intelligence':ab,ti OR 'ai':ab,ti OR 'wearable*':ab,ti
#4 'randomized controlled trial'/exp OR 'randomized controlled trial':ab,ti OR 'random*':ab,ti OR 'placebo':ab,ti OR 'clinical trial':ab,ti OR 'rct':ab,ti
#5 #1 AND #2 AND #3 AND #4

4. Cochrane Library:

search strategy:
#1 [mh "Aged"]
#2 ("aged" OR "older adult*" OR "older people" OR "elderly" OR "senior*" OR "aging"):ti,ab,kw
#3 #1 OR #2
#4 [mh "Tai Ji"]
#5 ("Tai Chi" OR "Taiji*" OR "Taichi" OR "Tai Chi Chuan" OR "Mind-body exercise"):ti,ab,kw
#6 #4 OR #5
#7 [mh "Virtual Reality"] OR [mh "Mobile Applications"] OR [mh "Telemedicine"] OR [mh "Telerehabilitation"]
#8 ("digital*" OR "virtual reality" OR "VR" OR "augmented reality" OR "AR" OR "mixed reality" OR "MR" OR "APP" OR "smartphone" OR "mobile health" OR "mHealth" OR "remote intervention" OR "tele-rehabilitation" OR "exergame*" OR "video game*" OR "sensor-based" OR "artificial intelligence" OR "AI" OR "wearable*"):ti,ab,kw
#9 #7 OR #8
#10 [mh "Randomized Controlled Trial"] OR [mh "Placebos"]
#11 ("randomized" OR "randomised" OR "randomly" OR "placebo" OR "clinical trial" OR "RCT"):ti,ab,kw
#12 #10 OR #11
#13 #3 AND #6 AND #9 AND #12

5. CINAHL:

search strategy:
#1 (MH "Aged+") OR (MH "Aging")
#2 TI ("aged" OR "older adults" OR "older people" OR "elderly" OR "senior*" OR "aging") OR AB ("aged" OR "older adults" OR "older people" OR "elderly" OR "senior*" OR "aging")
#3 #1 OR #2
#4 (MH "Tai Chi")
#5 TI ("Tai Chi" OR "Taiji*" OR "Taichi" OR "Tai Chi Chuan" OR "Mind-body exercise") OR AB ("Tai Chi" OR "Taiji*" OR "Taichi" OR "Tai Chi Chuan" OR "Mind-body exercise")
#6 #4 OR #5
#7 (MH "Virtual Reality") OR (MH "Mobile Applications") OR (MH "Telemedicine") OR (MH "Telerehabilitation") OR (MH "Artificial Intelligence")
#8 TI ("digital*" OR "virtual reality" OR "VR" OR "augmented reality" OR "AR" OR "mixed reality" OR "MR" OR "APP" OR "smartphone" OR "mobile health" OR

"mHealth" OR "remote intervention" OR "tele-rehabilitation" OR "exergame*" OR "video game*" OR "sensor-based" OR "artificial intelligence" OR "AI" OR "wearable*") OR AB ("digital*" OR "virtual reality" OR "VR" OR "augmented reality" OR "AR" OR "mixed reality" OR "MR" OR "APP" OR "smartphone" OR "mobile health" OR "mHealth" OR "remote intervention" OR "tele-rehabilitation" OR "exergame*" OR "video game*" OR "sensor-based" OR "artificial intelligence" OR "AI" OR "wearable*")

#9 #7 OR #8

#10 (MH "Randomized Controlled Trials") OR (MH "Placebos")

#11 TI ("randomized" OR "randomised" OR "randomly" OR "placebo" OR "clinical trial" OR "RCT") OR AB ("randomized" OR "randomised" OR "randomly" OR "placebo" OR "clinical trial" OR "RCT")

#12 #10 OR #11

#13 #3 AND #6 AND #9 AND #12

6. Scopus:

search strategy:

#1 TITLE-ABS-KEY ("aged" OR "older adults" OR "older people" OR "elderly" OR "senior*" OR "aging")

#2 TITLE-ABS-KEY ("Tai Chi" OR "Taiji*" OR "Taichi" OR "Tai Chi Chuan" OR "Mind-body exercise")

#3 TITLE-ABS-KEY ("digital*" OR "virtual reality" OR "VR" OR "augmented reality" OR "AR" OR "mixed reality" OR "MR" OR "APP" OR "smartphone" OR "mobile health" OR "mHealth" OR "remote intervention" OR "tele-rehabilitation" OR "exergame*" OR "video game*" OR "sensor-based" OR "artificial intelligence" OR "AI" OR "wearable*")

#4 TITLE-ABS-KEY ("randomized controlled trial" OR "random*" OR "placebo" OR "clinical trial" OR "RCT")

#5 #1 AND #2 AND #3 AND #4
